# Clinical Audits in Outpatient Clinics for Chronic Obstructive Pulmonary Disease: Methodological Considerations and Workflow

**DOI:** 10.1371/journal.pone.0141856

**Published:** 2015-11-06

**Authors:** Jose Luis López-Campos, María Abad Arranz, Carmen Calero Acuña, Fernando Romero Valero, Ruth Ayerbe García, Antonio Hidalgo Molina, Ricardo Ismael Aguilar Pérez-Grovas, Francisco García Gil, Francisco Casas Maldonado, Laura Caballero Ballesteros, María Sánchez Palop, Dolores Pérez-Tejero, Alejandro Segado, Jose Calvo Bonachera, Bárbara Hernández Sierra, Adolfo Doménech, Macarena Arroyo Varela, Francisco González Vargas, Juan Jose Cruz Rueda

**Affiliations:** 1 Unidad Médico-Quirúrgica de Enfermedades Respiratorias, Instituto de Biomedicina de Sevilla (IBiS), Hospital Universitario Virgen del Rocío/Universidad de Sevilla, Seville, Spain; 2 CIBER de Enfermedades Respiratorias (CIBERES), Instituto de Salud Carlos III, Madrid, Spain; 3 Hospital Puerta del Mar, Cádiz, Spain; 4 Hospital Juan Ramón Jiménez, Huelva, Spain; 5 Hospital Universitario Reina Sofía, Córdoba, Spain; 6 Hospital Universitario San Cecilio, Granada, Spain; 7 Hospital Infanta Margarita, Cabra, Córdoba, Spain; 8 Hospital Torrecárdenas, Almería, Spain; 9 Hospital Regional Universitario de Málaga, Spain; 10 Hospital Universitario Virgen de las Nieves, Granada, Spain; New York University School of Medicine, UNITED STATES

## Abstract

**Objectives:**

Previous clinical audits for chronic obstructive pulmonary disease (COPD) have provided valuable information on the clinical care delivered to patients admitted to medical wards because of COPD exacerbations. However, clinical audits of COPD in an outpatient setting are scarce and no methodological guidelines are currently available. Based on our previous experience, herein we describe a clinical audit for COPD patients in specialized outpatient clinics with the overall goal of establishing a potential methodological workflow.

**Methods:**

A pilot clinical audit of COPD patients referred to respiratory outpatient clinics in the region of Andalusia, Spain (over 8 million inhabitants), was performed. The audit took place between October 2013 and September 2014, and 10 centers (20% of all public hospitals) were invited to participate. Cases with an established diagnosis of COPD based on risk factors, clinical symptoms, and a post-bronchodilator FEV_1_/FVC ratio of less than 0.70 were deemed eligible. The usefulness of formally scheduled regular follow-up visits was assessed. Two different databases (resources and clinical database) were constructed. Assessments were planned over a year divided by 4 three-month periods, with the goal of determining seasonal-related changes. Exacerbations and survival served as the main endpoints.

**Conclusions:**

This paper describes a methodological framework for conducting a clinical audit of COPD patients in an outpatient setting. Results from such audits can guide health information systems development and implementation in real-world settings.

## Introduction

Despite the availability of guidelines, there is evidence that several diseases are frequently managed in a manner that is not in line with recommendations [1]. To a degree, this may reflect variations in clinical presentation, patient characteristics, and/or limited awareness of recommendations [2]. In this context, the critical analysis of medical records through an audit may prove useful in amending clinical practice [3, 4], with the ultimate goal of improving patient outcomes [5].

Clinical audits to assess the quality of care delivered and the attainment of treatment goals are paramount for the management of various chronic diseases, including chronic obstructive pulmonary disease (COPD). COPD is a major public health problem, greatly impacting morbidity and mortality [6], quality of life [7], and healthcare costs [8]. In this scenario, audits represent powerful tools for the creation of positive changes and improvements in COPD management.

Clinical audits in the field of COPD have been implemented in the United Kingdom [9], Spain [10, 11], and several other countries [2, 12, 13]. Recently, a European Clinical COPD Audit has been carried out through 13 European countries [14, 15]. The results of these in-hospital audits have provided valuable information on the clinical care provided to patients admitted to medical wards because of COPD exacerbations [15], as well as the available resources [16] and the interrelationships between health resources and clinical practice [17]. Unfortunately, clinical audits of COPD in an outpatient setting are scarce [18] and no general methodological guidelines are currently available. Insight into the success rate of implementation of guidelines and recommendations for COPD on an outpatient basis has the potential to improve long-term outcomes. Based on our previous experience in the field [11], herein we describe a clinical audit for COPD patients in specialized outpatient clinics with the overall goal of establishing a potential methodological workflow. The results for the current study can guide health information systems development and implementation in real-world settings.

## Methodology

### Audit implementation

We conducted a pilot clinical audit of COPD patients referred to respiratory outpatient clinics in the region of Andalusia, Spain (eight provinces with over 8 million inhabitants). The principal investigator and a project manager (both pulmonologists with expertise in auditing) were responsible of the design of the audit and periodically supervised the conduct of research (including research meetings and on-demand requests). In each center, there were two investigators: a local investigator (in charge of supervising the audit procedures, obtaining permissions, and coordinating the project) and a local data manager (in charge of recruiting cases and collecting data).

### Selection of hospitals

Andalusian public hospitals are divided into four categories (Table 1). The goal of the current audit was to include 20% of all public hospitals located in the region (n = 47). Consequently, a total of 10 hospitals were invited to participate. Randomization was not performed because we did not aim to achieve a representative sampling. The selection of candidate hospitals was based on the following two criteria: 1) participation in previous in-hospital audits, and 2) expression of interest after an initial proposal of the audit. Both large university hospitals and small community-based centers with respiratory outpatient clinics were considered for inclusion. However, participation rates were higher for regional hospitals (Table 1).

**Table 1:** Types of hospitals in Andalusia and their participation in the audit.

Type of hospital	Description	Number of centers	Number of centers invited to the audit
Regional hospitals	Highly ranked hospitals offering all medical specialties and providing care to the entire regional population.	9	7
Specialty hospitals	Hospitals offering a higher number of medical specialties than district hospitals and providing care to the entire province in which they are located.	8	2
District hospitals	Hospitals offering basic medical specialties and providing care to the population of the town in which they are located (including close villages located at a maximum of 1 hour away).	17	1
High-resolution hospitals	Small community-based hospitals providing joint consultation and treatment and equipped with a limited number of beds for short stays.	13	0

### Inclusion and exclusion criteria

Cases with an established diagnosis of COPD based on risk factors, clinical symptoms, and a post-bronchodilator FEV_1_/FVC ratio of less than 0.70 were deemed eligible [19]. Because our goal was to assess the usefulness of formally scheduled regular follow-up visits, only cases with at least 1 year of follow-up were included in the audit. Patients who underwent a first diagnostic visit or presented with an exacerbation were not eligible. Similarly, subjects with significant comorbidities that could have an impact on the COPD treatment approach were excluded at the local investigator’s discretion. Based on our previous experience, we estimated that 80 cases per center would be required for this pilot study. As of September 2013, assessments were planned over a year divided by 4 three-month periods (i.e., trimesters), with the goal of determining seasonal-related changes. Investigators were instructed to identify consecutive COPD cases at the beginning of each trimester until the desired sample size of 20 was reached. Enrollment was aimed at achieving a male to female ratio of 1:1 and including a proportionate distribution of COPD severity stages according to the degree of spirometric impairment (Table 2).

**Table 2:** Cases included in the audit according to the enrollment period, sex, and COPD severity stages.

	Fourth trimester 2013 (M:F)	First trimester 2014 (M:F)	Second trimester 2014 (M:F)	Third trimester 2014 (M:F)	Total (M:F)
GOLD 1	5 (3:2)	5 (2:3)	5 (3:2)	5 (2:3)	20 (10:10)
GOLD 2	5 (3:2)	5 (2:3)	5 (3:2)	5 (2:3)	20 (10:10)
GOLD 3	5 (3:2)	5 (2:3)	5 (3:2)	5 (2:3)	20 (10:10)
GOLD 4	5 (3:2)	5 (2:3)	5 (3:2)	5 (2:3)	20 (10:10)
Total	20 (12:8)	20 (8:12)	20 (12:8)	20 (8:12)	80 (40:40)

Abbreviations: M:F = males:females; GOLD = Global Initiative for Obstructive Lung Disease.

### Data collection

The audit was based on two different datasets, i.e., the resources database and the clinical database. The resources database included information on resources and organization of outpatient care in each participating center. This dataset was compiled one time for each hospital before starting the collection of clinical data. All COPD patient data were included in the clinical database. The variables to be included in the clinical database were proposed by the study coordinators and then discussed via e-mail and subsequently in a face-to-face meeting (held on July 2013 in Antequera, Málaga, Spain) with all of the investigators. The methodological aspects of the audit were thoroughly discussed during the meeting. Finally, a standardized questionnaire including 55 variables for the resources database and 182 variables for the clinical database was developed. The resources variables comprised the following information: 1) type and size of the center, 2) organization and performance of the outpatient facilities, 3) complementary diagnostic tools available for routine use, and 4) availability of specific COPD treatments. The clinical variables included 1) personal data, 2) general medical history, 3) history of COPD, 4) current clinical status, 5) complementary studies, 6) complete diagnostic information, and 7) assigned treatment.

### Web-based data entry system

A web-based data entry and management system was specifically developed for the audit by a quality freelance programmer. Access to the database was hierarchical, with local researchers having access to their own cases only and the study coordinators having the ability to retrieve and download all of the study data. Moreover, a computer program was made available to the coordinators for converting data directly into an SPSS system file format suitable for statistical analysis.

### Study protocol

The 1-year audit took place between October 2013 and September 2014. Recruitment was performed along 4 three-month periods (October–December 2013, January–March 2014, April–June 2014, and July–September 2014). At the beginning of each period, investigators were instructed to review the list of patients attending their outpatient clinics and identify eligible COPD cases who met the inclusion criteria according to their medical history. After completion of the 1-year follow-up, the number of emergency room visits or acute hospital admissions for COPD exacerbations as well as data on the vital status were collected from the medical records. Results were not disclosed to the physicians being audited to avoid changes in their clinical practice. Moreover, they were unaware of the timeframe of the year being audited. When necessary, the principal investigator provided additional instructions on the general conduct of the audit as well as the methodology to collect the variables. Moreover, a dedicated telephone line was established to respond to questions arising from audit sites in a timely manner. The project manager was in charge of identifying missing values, outliers, and inconsistencies, requiring the participation of local investigators to correct these.

### Guidelines benchmarking

The performance of the outpatient clinics was benchmarked against clinical guidelines available at the time of the audit. Throughout the study period, two guidelines–including GOLD 2013 [19] and the Spanish National Guideline for COPD (GesEPOC) [20]–were widely and uniformly used in Spain. We therefore carefully reviewed the two guidelines to extract the main statements for the purpose of benchmarking the audited performances. We also considered the 2009 Spain Health-Care Quality Standards in COPD that were active at the time of the audit [21]. The guideline statements used for benchmarking are summarized in Tables 3–5. The appropriateness of the outpatient performances in relation to such statements was categorized as follows: excellent (>80%), good (60−80%), adequate (40−59%), inadequate (20−39%), and highly inadequate (<20%).

**Table 3:** Guidelines statements used for benchmarking the outpatient clinics.

Guideline	Statement
Clinical interview	
GOLD 2013	COPD assessment must consider the following aspects of the disease separately: current level of patient’s symptoms, severity of the spirometric abnormality, exacerbation risk, presence of comorbidities
GesEPOC 2012	The clinical phenotype of COPD should be established in all patients. GesEPOC sets four different clinical phenotypes: A) non-frequent exacerbator, with emphysema or chronic bronchitis; B) COPD-asthma overlap; C) emphysema frequent exacerbator; and D) chronic bronchitis frequent exacerbator.
GesEPOC 2012	The diagnosis of COPD is based on a decrease in the expiratory flow, measured by FEV_1_ and its ratio to the forced vital capacity (FEV_1_/FVC).
GOLD 2013	At each visit, inquire about changes in symptoms since the last visit, including cough and sputum, breathlessness, fatigue, activity limitation, and sleep disturbances.
GOLD 2013	GOLD recommends the use of the Modified British Medical Research Council (mMRC) questionnaire or the COPD Assessment Test (CAT).
GesEPOC 2012	The severity of a patient with COPD is determined by the BODE index. Alternatively, the BODEx index can be used for patients with mild-to-moderate COPD.
GOLD 2013	Comorbidities should be looked for routinely, and treated appropriately, in any patient with COPD.
GOLD 2013	At each visit, determine current smoking status and smoke exposure
GOLD 2013	Dosages of various medications, adherence to the regimen, inhaler technique, effectiveness of the current regime at controlling symptoms, and side effects of treatment should be monitored.
Complementary tests: spirometry	
GOLD 2013	Decline in lung function is best tracked by spirometry performed at least once a year to identify patients whose lung function is declining quickly
GOLD 2013	Spirometry should be performed after the administration of an adequate dose of a short-acting inhaled bronchodilator in order to minimize variability.
Complementary tests: chest X-ray	
GOLD 2013	A chest X-ray is not useful to establish a diagnosis in COPD, but it is valuable in excluding alternative diagnoses and establishing the presence of significant comorbidities
GesEPOC 2012	A chest X-ray should be performed for initial assessment and to rule out the presence of complications: sudden dyspnea of unexplained origin (pneumothorax), changes in the pattern of cough or hemoptysis (neoplasia), or suspected pneumonia.
Complementary tests: Computed tomography (CT)	
GOLD 2013	Computed tomography (CT) of the chest is not routinely recommended. However, when there is doubt about the diagnosis of COPD, CT scanning might help in the differential diagnosis where concomitant diseases are present.
GesEPOC 2012	Indications for a chest CT scan: frequent exacerbator phenotype for the diagnosis of bronchiectasis, exclusion of other associated lung diseases, diagnosis and evaluation of emphysema
Complementary tests: Lung volumes and diffusing capacity	
GOLD 2013	Lung Volumes and Diffusing Capacity help characterize the severity of COPD but are not essential to patient management
SEPAR 2009	Patients with severe or very severe COPD should undergo the following tests at least one time: measurement of static lung volumes and carbon monoxide diffusing capacity
GesEPOC 2012	Indication for lung volumes determination: suspected restrictive component or grades III–IV obstruction for investigating lung hyperinflation
GesEPOC 2012	Indication for diffusing capacity determination: grades III–IV obstruction, hypoxia or severe dyspnea not proportional to the degree of obstruction, investigation of emphysema
Complementary tests: Pulse oximetry and arterial blood gases analysis	
GOLD 2013	Pulse oximetry should be used to assess all stable patients with FEV_1_ <35% predicted or with clinical signs suggestive of respiratory failure or right heart failure
GesEPOC 2012	Pulse oximetry is useful in the evaluation of suspected hypoxemia, either in seriously ill patients or for the treatment of exacerbations
GOLD 2013	If peripheral saturation is <92% arterial blood gases should be assessed
GesEPOC 2012	Indications for arterial blood gas analysis: grades III–IV obstruction or FEV_1_ <1 L, MRC dyspnea 3–4, signs of pulmonary hypertension and/or cor pulmonale, indication and monitoring of patients with OCD, hematocrit >55%, cyanosis and/or pulse oximetry <92%
Complementary tests: Alpha1-antitrypsin	
GOLD 2013	The World Health Organization recommends that COPD patients from areas with a particularly high prevalence of alpha-1 antitrypsin deficiency should be screened for this genetic disorder
SEPAR 2009	Plasma α1-antitrypsin concentrations should be determined for all COPD patients at least one time
GesEPOC 2012	In all patients with COPD plasma concentration of alpha-1-antitrypsin should be determined at least one time
Complementary tests: Exercise test and physical activity	
GOLD 2013	Objectively measured exercise impairment, assessed by a reduction in self-paced walking distance or during incremental exercise testing in a laboratory, is a powerful indicator of health status impairment and predictor of prognosis
GOLD 2013	Monitoring of physical activity may be more relevant regarding prognosis than evaluating exercise capacity
SEPAR 2009	Patients with severe or very severe COPD should undergo the following test at least one time: maximal exercise test
GesEPOC 2012	Indications for 6-min walking test: calculate BODE index, obstruction grades III-IV, prior to the evaluation for respiratory rehabilitation
GesEPOC 2012	Indications for maximal exercise test: evaluation for pulmonary rehabilitation
Nutritional assessment	
SEPAR 2009	Patients with severe or very severe COPD should undergo the following test at least one time: nutritional assessment

GOLD 2013: Global Initiative for Obstructive Lung Disease 2013 [19].

SEPAR 2009: SEPAR Health-Care Quality Standards 2009 [21].

GesEPOC 2012: Spanish National Guidelines for COPD [22].

**Table 4:** Guidelines statements used for benchmarking non-pharmacological therapeutic options.

Guideline	Statement
Tobacco	
GOLD 2013	Health care providers are important for the delivery of smoking cessation messages and interventions and should encourage all patients who smoke to quit
GesEPOC 2012	It is recommended to offer all smokers with COPD advice to quit supported by medical/psychological counseling
Vaccinations	
GOLD 2013	Influenza vaccination can reduce serious morbidity (such as lower respiratory tract infections requiring hospitalization) and mortality in COPD patients
SEPAR 2009	Influenza vaccination should be recommended to all COPD patients
GOLD 2013	Pneumococcal polysaccharide vaccine is recommended for COPD patients aged 65 years and older, as well as in younger patients with significant comorbidities (e.g., cardiac diseases)
SEPAR 2009	Pneumococcal vaccination should be offered to patients with severe COPD and to all COPD patients aged 65 years and older
GesEPOC 2012	All patients with COPD should be vaccinated annually against influenza and should receive pneumococcal vaccine
Exercise	
GOLD 2013	All patients who get short of breath when walking on their own pace on level ground should be offered rehabilitation
GOLD 2013	Physical activity is recommended for all patients with COPD
SEPAR 2009	Regular exercise should be recommended to all COPD patients
SEPAR 2009	Pulmonary rehabilitation should be prescribed for all COPD patients who continue to experience limitations in activities of daily living because of dyspnea after stage-appropriate pharmacological treatment
GesEPOC 2012	Regular physical activity should be advised to all patients with COPD
Long-term oxygen therapy	
GOLD 2013	Long-term oxygen therapy is indicated for patients with the following characteristics: PaO_2_ ≤ 7.3 kPa (55 mmHg) or SaO2 ≤ 88%, with or without hypercapnia confirmed twice over a 3-week period (evidence B); or PaO_2_ between 7.3 kPa (55 mmHg) and 8.0 kPa (60 mmHg); or SaO_2_ >88% in presence of pulmonary hypertension, peripheral edema suggesting congestive cardiac failure, or polycythemia (hematocrit > 55%)
GesEPOC 2012	Long-term oxygen therapy is indicated for patients with stable COPD patients at rest at sea level, breathing air with the following characteristics: PaO_2_ <55 mmHg or PaO_2_ between 55 and 60 mmHg in presence of conditions that may increase the risk of hypoxemia, including hypertension pulmonary/cor pulmonale, congestive heart failure/arrhythmias, or hematocrit >55%.

GOLD 2013: Global Initiative for Obstructive Lung Disease 2013 [19].

SEPAR 2009: SEPAR Health-Care Quality Standards 2009 [21].

GesEPOC 2012: Spanish National Guidelines for COPD [22].

**Table 5:** Guidelines statements used for benchmarking pharmacological therapeutic options.

Guideline	Statement
Bronchodilators	
GOLD 2013	Bronchodilator medications are given on either an as-needed basis or a regular basis to prevent or reduce symptoms
GesEPOC 2012	Short-acting bronchodilators (β2 agonists and/or anticholinergic drugs) should be used on demand for immediate relief in patients with COPD as an add-on to basal treatment regardless of disease severity
GesEPOC 2012	Long-acting bronchodilators should be used as first-line treatment in all patients with chronic symptoms
GesEPOC 2012	Tiotropium should preferred to salmeterol in patients with stable COPD who require a sustained action bronchodilator as monotherapy and had experienced at least one previous exacerbation requiring hospitalization and/or treatment with systemic corticosteroids and/or antibiotics during the previous year
GesEPOC 2012	The choice of the bronchodilator in patients with stable COPD who require a sustained action bronchodilator as monotherapy should be based on 1) the patient’s preferences, 2) the individual response to the drug, and 3) pharmacoeconomic considerations
GesEPOC 2012	Combinations of long-acting bronchodilators should be considered for COPD patients with persistent symptoms despite monotherapy
Inhaled steroids combinations	
GOLD 2013	Long-term treatment with inhaled corticosteroids is recommended for patients with severe and very severe COPD and frequent exacerbations not adequately controlled by long-acting bronchodilators
GOLD 2013	Long-term monotherapy with inhaled corticosteroids is not recommended in COPD because it is less effective than the combination of inhaled corticosteroids with long-acting β2 agonists
GesEPOC 2012	Inhaled corticosteroids should invariably be used in association with long-acting bronchodilators
GesEPOC 2012	Combinations of long-acting β2 agonist and inhaled corticosteroids should be used in patients with COPD who present frequent exacerbations despite treatment with long-acting bronchodilators
GesEPOC 2012	Triple therapy (addition of a long-acting antimuscarinic agent to a long-acting β2 agonist and inhaled corticosteroids) should be used in patients with severe or very severe COPD and poorly controlled symptoms despite treatment with long-acting bronchodilators
SEPAR 2009	Triple therapy (addition of a long-acting antimuscarinic agent to a long-acting β_2_ agonist and inhaled corticosteroids) is justified in patients with severe or very severe COPD in presence of symptomatic deterioration despite treatment with long-acting bronchodilators
Oral steroids	
GOLD 2013	Long-term monotherapy with oral corticosteroids is not recommended in COPD
SEPAR 2009	Oral corticosteroids are not recommended for maintenance therapy in stable COPD
Antibiotics	
GOLD 2013	The use of antibiotics (other than for treating infectious exacerbations of COPD and other bacterial infections) is not currently indicated
GesEPOC 2012	The coexistence of COPD with frequent exacerbations and bronchiectasis with chronic bronchial infection should be treated with antibiotics, in line with the recommendations for bronchiectasis
Mucolytics	
GOLD 2013	Mucolytics: the widespread use of these agents cannot be recommended at present
GOLD 2013	There is some evidence that treatment with mucolytics (such as carbocysteine and N-acetyl-cysteine) may reduce exacerbations in COPD patients not receiving inhaled corticosteroids
GesEPOC 2012	Carbocysteine is suggested as a maintenance treatment in patients with stable COPD, an exacerbator phenotype, and chronic bronchitis
Other treatments	
GOLD 2013	The phosphodiesterase-4 inhibitor, roflumilast, may also be used to reduce exacerbations in patients with chronic bronchitis, severe and very severe COPD, and frequent exacerbations not adequately controlled by long-acting bronchodilators
GOLD 2013	Young patients with severe hereditary alpha-1 antitrypsin deficiency and established emphysema may be candidates for alpha-1 antitrypsin augmentation therapy
GOLD 2013	Nedocromil and leukotriene modifiers have not been adequately tested in COPD patients and cannot be recommended
Methylxanthines	
SEPAR 2009	Theophyllines should be used whenever a patient remains symptomatic despite appropriate treatment according to disease stage, or in the few cases in which an oral route is required
GesEPOC 2012	Theophylline should not be used as first-line treatment because of its potential adverse effects
GOLD Treatment strategies	
GOLD 2013	Group A. A short-acting bronchodilator is recommended as first choice for all Group A patients
GOLD 2013	Group A. A combination of short-acting bronchodilators or the introduction of a long-acting bronchodilator represents the second choice
GOLD 2013	Group B. Long-acting bronchodilators are superior to short-acting bronchodilators (taken as needed or as necessary) and are therefore recommended
GOLD 2013	Group B. The second choice for patients with severe breathlessness is a combination of long-acting bronchodilators
GOLD 2013	Group C. A fixed combination of inhaled corticosteroid/long-acting beta2-agonist or a long-acting anticholinergic drug is recommended as first choice
GOLD 2013	Group C. A combination of two long-acting bronchodilators or the combination of inhaled corticosteroid/long-acting anticholinergic can be use as second choice
GOLD 2013	Group D: The first choice consists of an inhaled corticosteroid plus a long-acting β2 agonist or a long-acting anticholinergic drug, with some evidence for triple therapy
GOLD 2013	Group D. A combination of all three classes of drugs (inhaled corticosteroids/long-acting β2 agonist/long-acting anticholinergic drugs) can be used as second choice although conflicting data exist
GesEPOC Treatment strategies	
GesEPOC 2012	The drugs to be added to long-acting bronchodilators depend on the clinical phenotype. The treatment of the non-exacerbator phenotype in emphysema or chronic bronchitis is based on the use of combined long-acting bronchodilators
GesEPOC 2012	The treatment of patients with an overlapping phenotype is based on the use of long-acting bronchodilators combined with inhaled corticosteroids
GesEPOC 2012	The treatment of the frequent exacerbator phenotype with emphysema is based on long-acting bronchodilators, with the potential addition of inhaled corticosteroids and theophylline according to the severity level
GesEPOC 2012	The treatment of the chronic bronchitis frequent exacerbator phenotype is based on long-acting bronchodilators, with the potential addition of inhaled corticosteroids, phosphodiesterase 4 inhibitors or mucolytics according to the severity level. In selected cases, the preventive use of antibiotics can be considered

GOLD 2013: Global Initiative for Obstructive Lung Disease 2013 [19].

SEPAR 2009: SEPAR Health-Care Quality Standards 2009 [21].

GesEPOC 2012: Spanish National Guideline for COPD [22].

### Ethical statement

The audit was approved by the Ethics Committee of the Hospital Universitario Virgen del Rocío (code: 2013PI/201). Clinical records were anonymized in the database by assigning a numerical code through an algorithm. No personal information was registered that could be used directly or indirectly to identify an individual. The relationship between the audit code and the clinical history number was kept locally under the local investigator’s responsibility. Because of the retrospective nature of the study, the annonimization of data, and the lack of active research interventions, the need for informed consent was waived. The Ethics Committee was aware of this circumstance, clearly explained in the protocol, and approved this procedure.

### Statistical analysis

All computations were performed using the Statistical Package for Social Sciences, version 20.0 (SPSS; IBM Corporation, Somers, NY, USA). Clinical variables are presented as means and standard deviations or absolute and relative frequencies, as appropriate. Quantitative variables were analysed using the unpaired Student’s *t*-test for independent data, preceded by the Levene test to evaluate homogeneity of variances. Categorical data were compared using the χ^2^ test. The variables associated with exacerbations and mortality during the 1-year follow-up were identified using hierarchical multilevel multivariate logistic regression analysis, as previously described [23]. The alpha error was set at 0.05.

## Discussion

This paper describes a methodological framework for conducting a clinical audit of COPD patients in an outpatient setting. Because of the substantial burden of COPD on both patients and healthcare systems, the lack of implementation of evidence-based guidelines in clinical practice remains a major concern for professional respiratory societies [24]. In this context, clinical audits may have an important role in the drive to improve the quality of COPD care. Outpatient-based clinical audits have been previously performed in different disease conditions [25–27]. However, most audits in the field of respiratory diseases have been conducted in an in-hospital scenario [15]. Clinical audits of COPD in an outpatient setting are scarce [28, 29] and no methodological guidelines are currently available. In 2011, the ALT-BPCO (Appropriateness of Long Term treatment of COPD) project has been launched with the overall goal of auditing the applicability of COPD guidelines in an Italian region (Campania) and identifying potential obstacles to their implementation [18]. The audit was conducted in 29 respiratory units (65.8% of the total regional units), with patients being followed-up for 6 months. The authors identified significant gaps between current practice and guideline recommendations with regard to both medical practice (mean agreement: 25%) and health organization (mean agreement: 48%). Of note, significant improvements were observed after 6 months both in terms of clinical practice (60.7%) and organization (54.7%) [18].

Genuine auditing demands more comprehensive efforts than simply recording and analyzing clinical information. In this regard, the overall goal of clinical audits is to improve the quality of care through its systematic assessment against a defined standard [30], with all levels of staff being involved in reviewing activities [4, 11]. A number of methodological considerations need to be taken into account when planning an outpatient clinical audit. The main limitation of this audit lies in its lack of representativeness. Participating facilities should be representative (both in terms of type and number) of all centers located in the area under investigation. Because of the pilot nature of the current audit, we did not aim to obtain a representative sample of all Andalusian outpatient facilities for COPD. In general, large hospitals (overrepresented in the current study) may have more resources in place for delivering better clinical care [31]. However, the European COPD audit demonstrated that hospital size was positively related to clinical performances only for a minority of indicators and that differences in guideline adherence in relation to hospital size were lower than expected [16]. Nonetheless, auditing a random selection of outpatient centers for COPD will be paramount in the future because differences in guidelines adherence alone are not sufficient to explain the variance in COPD care [32]. Another limitation of the present auditing protocol is its focus on the cross-sectional evaluation of a single, specific clinical visit. Such an approach allowed assessing the performance of only one doctor during a routine clinical visit. It is also expected that former clinical acts could have had an impact on the visit under investigation (e.g., lack of spirometry if already recently performed). These limitations should be kept in mind when the final results will be available. Because of its pilot nature, the number of collected variables was extensive but not comprehensive. Notably, the audit was designed based on our previous experience. These caveats notwithstanding, the current report will be helpful to guide the selection of the study variables in future, larger audits being performed in an outpatient setting.

The potential clinical applicability of the current protocol was carefully considered when the audit was designed. Clinical audits are aimed at summarizing the clinical performance of healthcare over a specified period of time. In general, they allow health professionals to assess and adjust their performance. We are confident that the proposed protocol can be implemented in a prompt manner. To this aim, we believe it is important that investigators and/or their representative sampling are implicated in the following milestones that should be achieved in real practice: 1) the conception of the audit, 2) determination of the temporal coverage, 3) selection of variables to be collected, and 4) identification of the main clinical outcome measures.

The accuracy of an audit is generally dependent on the persons responsible for the collection of data. Although auditors external to the organization being audited could maintain a neutral, unbiased attitude, they are not normally experts in the field under investigation. Moreover, the use of external auditors leads to increased audit costs and imposes a heavier legal burden for data access rights. In turn, internal auditors spend most of their time working in the clinical structure being audited; as a result, they have a better understanding of the culture, workflows and the clinical relevance of data. However, their expertise in auditing may be limited and policies to maintain internal auditors’ objectivity should be implemented. In the present study, we relied on a selected number of internal auditors (pulmonologists with expertise in COPD) who participated in previous audits. In the initial stages of study implementation, the study protocol was thoroughly reviewed in a joint investigator meeting. In addition, adequate supervision was provided throughout the audit and results were not disclosed to the physicians being audited to avoid changes in their clinical practice. Because recruitment was performed along 4 three-month periods, auditors were unaware of the timeframe of the year under investigation. Finally, the personnel in charge of data analysis were external to the participating outpatient clinics.

The source of information to be used in clinical audits remains a matter of debate. The current audit was based on a retrospective review of prospectively included cases extracted from medical records. The two main consequences of this approach include 1) the occurrence of missing values and 2) the heterogeneity of clinical documents and sources (e.g., medical charts, nurses’ notes, administrative databases) used for data extraction [23]. One possible solution to this problem would lie in the use of electronic health records [33]. Because improvements in the quality of the underlying data lead to more reliable audit results, a careful data cleaning (aimed at keeping the number of errors and missing data as low as possible and gather maximum data for analysis) was performed in our study.

The number of candidate variables to be gathered and the choice of benchmark also pose significant methodological challenges for clinical audits [30]. Concerning the problem of variable selection, investigators should balance the inclusion of all of the useful variables with the efficiency of data analysis and the potential increases in statistical complexity and computation times. In turn, the selection of statements used for benchmarking should be informed by guidelines currently implemented in a specific geographic area (e.g., Spain for the current audit) [19, 21, 22].

Finally, an adequate data management is a critical phase for the generation of reliable conclusions from clinical audits. Quality control of data processing should be undertaken at regular intervals to minimize missing data, inconsistent data, and deviations from the protocol. Discrepancy management should include joint reviewing of discrepancies before declaring them as irresolvable. In addition, most of the data used for clinical audits are characterized by a hierarchical (multilevel) structure. Because standard multivariate regression methods tend to ignore this aspect (resulting in a loss of statistical efficiency and, in some cases, wrong conclusions), multilevel modeling is strongly recommended when hierarchical data structures are present [34]. Although the impact of a hierarchical structure is deemed to be minor when sample sizes are small (as in the current audit), multilevel modeling will be applied to identify variables significantly associated with outcomes in a follow-up study [23].

In summary, herein we have provided a methodological framework for conducting a clinical audit of COPD patients in an outpatient setting. Challenges to be considered when auditing current practice against a defined standard include a careful assessment of both patients’ characteristics and physicians’ attitude and knowledge. Hopefully, the methodology proposed in the current paper can guide health information systems development and implementation in real-world settings, with the ultimate goal of supporting a process of continuous quality improvement for COPD care.
